# Cannabidiol for Oral Health: A New Promising Therapeutical Tool in Dentistry

**DOI:** 10.3390/ijms24119693

**Published:** 2023-06-02

**Authors:** Luigi Bellocchio, Assunta Patano, Alessio Danilo Inchingolo, Francesco Inchingolo, Gianna Dipalma, Ciro Gargiulo Isacco, Elisabetta de Ruvo, Biagio Rapone, Antonio Mancini, Felice Lorusso, Antonio Scarano, Giuseppina Malcangi, Angelo Michele Inchingolo

**Affiliations:** 1 INSERM, U1215 NeuroCentre Magendie, Endocannabinoids and Neuroadaptation, University of Bordeaux, 33063 Bordeaux, France; 2 Department of Interdisciplinary Medicine, University of Study “Aldo Moro”, 70124 Bari, Italy; assuntapatano@gmail.com (A.P.); ad.inchingolo@libero.it (A.D.I.); giannadipalma@tiscali.it (G.D.); drciroisacco@gmail.com (C.G.I.); studio.deruvo@libero.it (E.d.R.); biagiorapone79@gmail.com (B.R.); dr.antonio.mancini@gmail.com (A.M.); giuseppinamalcangi@libero.it (G.M.); angeloinchingolo@gmail.com (A.M.I.); 3 Department of Medical, Oral and Biotechnological Sciences, University of Chieti-Pescara, 66100 Chieti, Italy; drlorussofelice@gmail.com (F.L.); ascarano@unich.it (A.S.)

**Keywords:** plants, cannabinoids, CBD, pharmacology, dental medicine, patents

## Abstract

The medical use of cannabis has a very long history. Although many substances called cannabinoids are present in cannabis, Δ9tetrahydrocannabinol (Δ9-THC), cannabidiol (CBD) and cannabinol (CBN) are the three main cannabinoids that are most present and described. CBD itself is not responsible for the psychotropic effects of cannabis, since it does not produce the typical behavioral effects associated with the consumption of this drug. CBD has recently gained growing attention in modern society and seems to be increasingly explored in dentistry. Several subjective findings suggest some therapeutic effects of CBD that are strongly supported by research evidence. However, there is a plethora of data regarding CBD’s mechanism of action and therapeutic potential, which are in many cases contradictory. We will first provide an overview of the scientific evidence on the molecular mechanism of CBD’s action. Furthermore, we will map the recent developments regarding the possible oral benefits of CBD. In summary, we will highlight CBD’s promising biological features for its application in dentistry, despite exiting patents that suggest the current compositions for oral care as the main interest of the industry.

## 1. Introduction

The use of *Cannabis sativa* L. for medical purposes dates back to an Egyptian medical papyrus (circa 1550 BC) [1]. Among the multitude of cannabinoids present in this plant, Δ^9^tetrahydrocannabinol (Δ9-THC), cannabidiol (CBD) and cannabinol (CBN) are the three main cannabinoids that are the most present and the best described components due also to their significant presence [2,3]. Cannabis is used in three different forms with different THC concentrations: marijuana, hashish, and hash oil [4].

**Figure 1:**
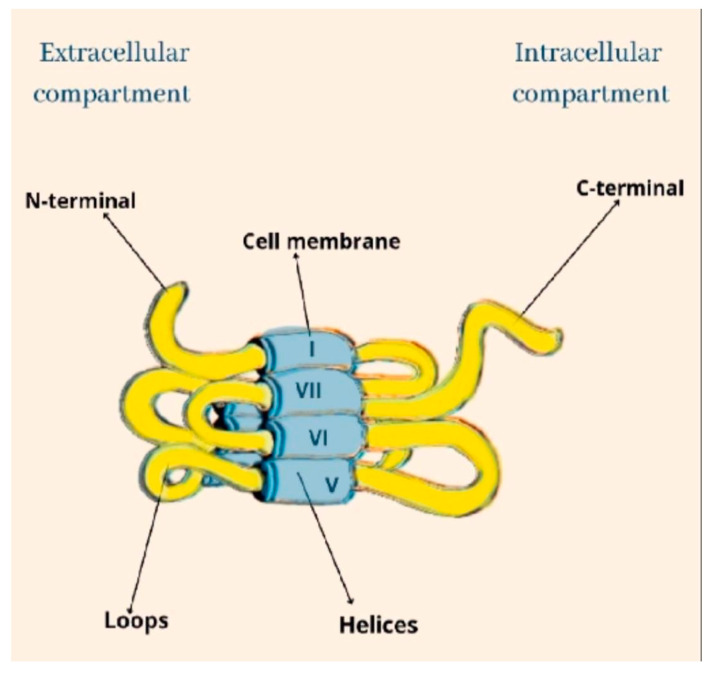
The structural architecture of CB.

Cannabinoids recognize and bind to specific receptors, the main ones being recognized in the CB_1_ and CB_2_ receptors. They are G-protein-coupled. Their polypeptide chain crosses the cell membrane seven times. The amine end remains on the extracellular side, while the carboxyl end remains on the intracellular side. They are characterized by three extracellular loops and three intracellular loops (Figure 1).

**Figure 2:**
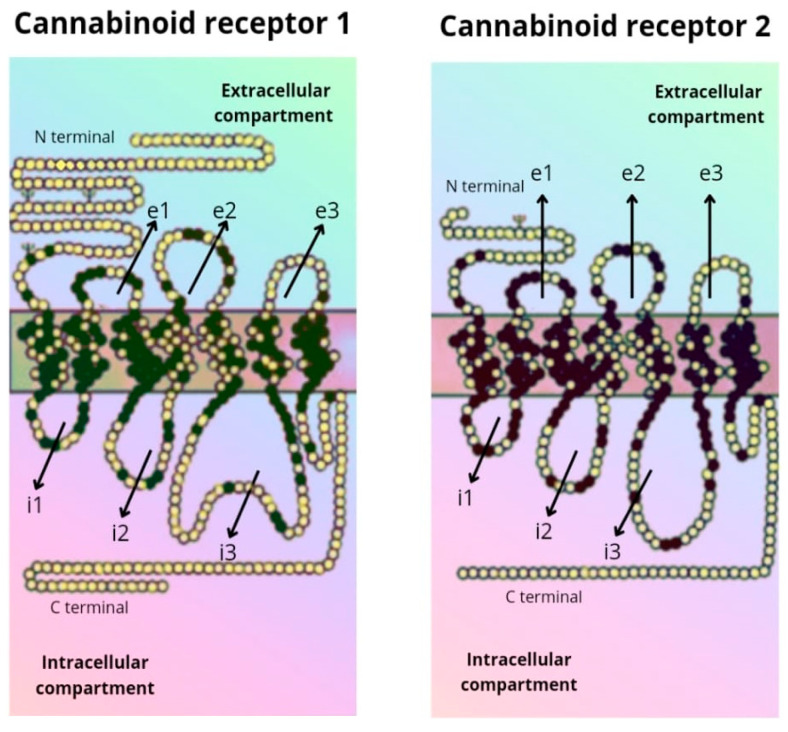
Amino acid sequence of the CB_1_ and CB_2_ receptor.

The CB_1_ receptor consists of a longer polypeptide chain than CB_2_ (472 amino acids in CB_1_, and 360 amino acids in CB_2_). The amino-terminal (extracellular) domain of CB_2_ is shorter. The complete amino acid sequence of the two receptors is homologous in 44% of them, while in the transmembrane domains the sequence is equal in 68% of them [5,6] (Figure 2).

Another receptor belonging to the GPCR family that binds to ECs is the G-protein coupled receptor 55 (GPR55), also known as CB3. It is supposed to modulate memory, motor activity, and cognitive function because of its high expression in the brain, particularly in the cerebellum [7,8]. At the peripheral level, GPR55s, being present in osteoblasts and osteoclasts, would modulate bone metabolism [9].

Widely present in humans is GPR119, which has been shown to represent another cannabinoid receptor that is encoded by the GPR119 gene [8]. It is present predominantly found in pancreatic (beta cells) and gastrointestinal cells. Recent studies attributed to GPR119’s therapeutic effects on diabetes and obesity highlight its direct action on insulin release in the pancreatic cells and indirectly at the level of intestinal enteroendocrine cells on the production of glucagon-like peptide 1 (GLP-1) [10,11].

Δ9-THC is the main psychoactive principle of cannabis and is known as the canonical agonist of both cannabinoid receptors, namely the CB_1_ and CB_2_ receptors, but with a relatively higher intrinsic affinity for CB_1_ than for CB_2_. THC is a hydrophobic and lipophilic compound [12,13]. Thus, many studies have been performed focusing on the pharmacology, therapeutic potential, and toxicity of Δ9-THC as a classical cannabinoid molecule in the last 70 years. These studies promoted the discovery and characterization of the endocannabinoid system (ECS) [14]. The ECS is made up of G-protein coupled (GPCR) cannabinoid receptors (CB_1_ and CB_2_) and their endogenous ligands, anandamide (AEA) and 2-arachidonoylglycerol (2AG), which fall into the category of endocannabinoids (ECs) [14]. In addition to the CB_1_ and CB_2_ receptors, the ECS also includes the peroxisome proliferator-activated receptor alpha (PPARα), GPR119, GPR55, and the transient receptor potential vanilloid 1 (TRPV1) receptors [15]. ECs are metabolized by multiple specific and non-specific enzymes. Those in the former category include fatty acid amide hydrolase (FAAH, for metabolism of AEA) and monoacylglycerol lipase (MAGL, for metabolism of 2-AG) [16]. Interestingly, since ECs share many structural similarities with prostaglandins, several interactions have between shown between the metabolic pathways for endocannabinoids and inflammatory lipids, including lipoxygenases and cyclooxygenases [16]. 

**Figure 3:**
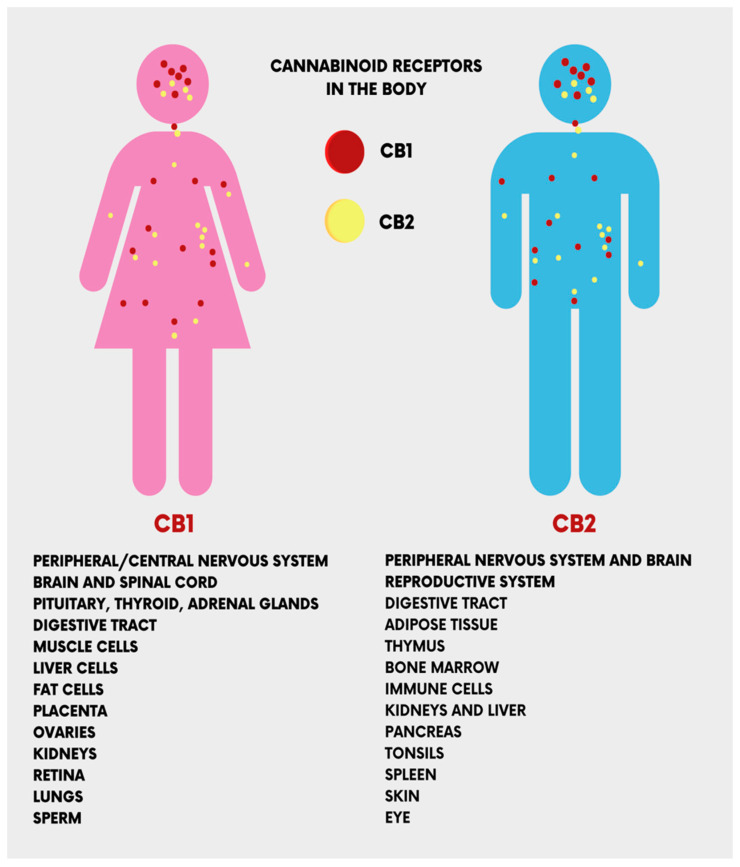
The location of the cannabinoid receptors (CB_1_ and CB_2_) in the human body.

The CB_1_ and CB_2_ receptors, encoded by the *CNR1* and *CNR2* genes, respectively, are the main receptors of ECs. They have different functionalities despite sharing more than 44% of amino acid sequences [17]. CB_1_ receptors are present in the central nervous system (the cerebellum, cerebral cortex, hippocampus, etc.), and act on cognitive functions, including memory, locomotion, and pain. At the peripheral level, CB_1_ receptors are present in multiple locations, cardiac cells, lung cells, immune cells, reproductive tissues, gastrointestinal system tissues, in the ganglia of the sympathetic nervous system, in the urinary bladder, and in adrenal gland cells, where their functions have been recognized but not well defined [18,19]. Peripherally, CB_2_ receptors are localized in monocytes/macrophages and poly-morphonuclear neutrophils, lymphocytes and natural killer cells in the testis, skeleton, liver and spleen. In the central nervous system (CNS), CB_2_ receptors are localized in the hippocampus and substantia nigra and the neuronal, glial, and endothelial cells of the cortex. At the CNS level, the functions of the CB_2_ receptor are not yet clear, but it is assumed that it may affect the neuro-immunological system [19] (Figure 3).

CBD has been isolated and described earlier than Δ9-THC [20]. However, it has remained a less studied molecule of cannabis, forgetting its participation in the psychotropic effects of this plant, since CBD use is not associated with the typical behavioral effects of cannabinoids [21]. CBD is a unisomer of THC, and is known to have a better effect on anxiety, cognition, and pain, with little psychoactivity. Compared with THC, CBD has a better affinity for CB_1_ and CB_2_ receptors, with the predominance of the latter, and could also interfere with the activity of THC [22,23,24].

Cannabinoids are mainly synthetized as acidic forms (A), thus Δ9-THC(A) and CBD(A) are the end-products of the enzymatic biosynthesis of cannabinoids. When exposed to heat (pyrolysis during smoking or baking), radiation, or spontaneously during storage, the compounds undergo decarboxylation and ‘spontaneous rearrangement’ reactions [25]. *C. sativa* accumulates THC and CBD in glandular trichomes in the aerial parts of the plant, but not on the root surface. Upon trichome thermal or mechanical ruptures, its contents form a sticky coating on the plant surface due to the viscous, non-crystallizing properties of cannabinoids, which will protect the plant from desiccation and/or potential herbivores [26]. The amount of cannabinoids formed in the trichomes correlates positively with increased temperatures and imposed heat stress, as well as with low soil moisture and poor mineral nutrient content [27]. Cannabinoid production may also provide an evolutionary advantage by functioning as sunscreens that absorb biologically destructive UV-B radiation (280–315 nm), as significantly increased cannabinoid production was measured in cannabis flowers after UV-B-induced stress [28]. Furthermore, cannabinoids in general [29], and CBD in particular [30], have a significant antimicrobial action, which confers high climate resistance and soil adaptability to cannabis. Thus, phytocannabinoids convey various biologically beneficial properties for the plant.

**Figure 4:**
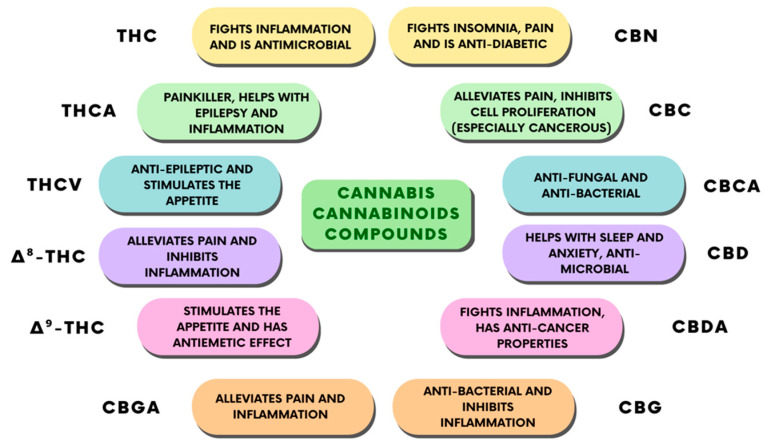
Classification of the different cannabinoid compounds derived from cannabis and their therapeutic properties.

The use of CBD has always represented a complicated legal issue worldwide, and this has often restricted the scientific studies and professional awareness about its therapeutic applications. Apart from this, numerous individual findings suggest some therapeutic effects of CBD, which have been reported to include antipsychotic, anticonvulsant, neuroprotective, anxiolytic, and sleep-promoting effects [31]. Furthermore, pre-clinical and clinical studies attributed a desirable safety profile to CBD [31], associated with its anti-inflammatory effects [32]. However, elucidating the pharmacodynamics of CBD has always proven to be difficult for scientists, beginning with initial reports in which CBD was shown to weakly bind to cannabinoid receptor orthosteric sites when compared to canonical agonists [33], indicating that CBD’s effects might be independent of the cannabinoid receptors. This conclusion was proved to be partially true by other studies, where researchers found a direct interaction of CBD with several receptors, enzymes and ion channels. Recently, however, some reports found both a direct and an indirect modulation of ECS activity from CBD [33]. Taken together, these findings point out CBD as a novel promising phytocannabinoid-based medicine. Indeed, the therapeutic uses of CBD are mostly linked to its anti-inflammatory, antioxidant, and analgesic properties [34]. Thus, CBD is endowed with many potential applications, such as in bone tissue processes [35,36], neuroprotection, epilepsy, anxiety, and cancer [37]. CBD also has some other effects that have not been fully studied, including relaxation, improved sleep, and stress relief, given edible, tincture, and vape formulations of the drug (Figure 4).

**Figure 5:**
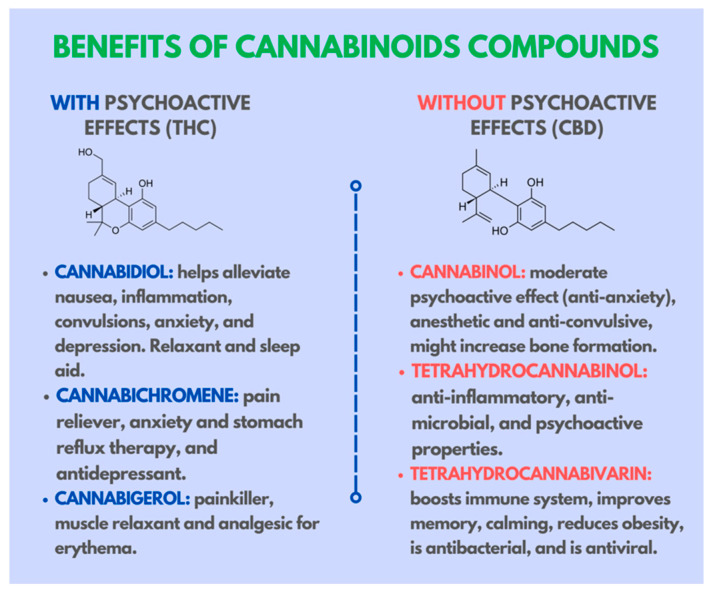
Benefits of cannabinoid compounds and their effects on the human body.

In recent years, thanks to various public and private institutions, much research and development of CBD have been performed, especially with regard to its therapeutic uses. Indeed, approximately USD 30 bilions is expected to be reached by the CBD market in 2025 [38]. Amongst the various potential uses of cannabinoids [39], and CBD in particular [40], dentistry and oral medicine have recently attracted greater attention [15]. In particular, studies have been exploring the possible medical applications of CBD use in the oral cavity [41], together with the functional and anatomical characterization of the ECS in this part of the body [15,41,42,43], in addition to its modulation by pathological status [15,44]. The aim of this narrative review is to provide an historical overview on cannabis use and the ECS, as well as to explore the mechanism of action of CBD and to summarize the recent scientific and technological discoveries of current CBD use and its possible future applications in the field of oral health (Figure 5).

### 1.1. Cannabis and the Endocannabinoid System in Human History 

Consumed in the form of Marijuana, hashish, or bhang, cannabis sativa extracts are the most widely used recreational drugs, with more than 200 million cannabis users worldwide (World Drug Report 2020, United Nations). Its recreational and therapeutic use are due to its psychoactive effects, amongst others, such as changes in sensory perception, relaxation, and euphoria [45]. However, the Cannabis Sativa plant is one of the first plants that was used by man for fiber, food, medicine, religious, or recreational contexts. The first reference to the use of cannabis as medicine comes from a Chinese pharmacological treatise attributed to Emperor Shen Nung (3000 BCE), which makes cannabis one of the recreational drugs with the longest recorded history of human use [45]. 

The medicinal use of cannabis was present in most ancient civilizations, and was used by the Assyrians, Egyptians, Greeks, and Romans. The Aryan and Indo-European populations who lived in ancient Iran and India (source Treccani.it, Britannica.com), used cannabis in their societies, and given their migrations in prehistoric times, they might have passed on their knowledge to other groups [46]. Cannabis had several applications: as a bandage for swelling and bruising; in fumes for arthritis; either as a drink or in the food for depression, for kidney stones, for impotence, and for annulling witchcraft. In ancient India, it was prepared in the form of a mild drink, called bhang, and it was described as an anti-anxiety drug thanks to its power “to free people from distress” (circa 1500 BCE) [46].

Ancient civilizations were aware of the dual nature of cannabis and its psychoactive proprieties, and some texts defined it as “the drug which takes away the mind”. Similarly, accounts of its nefarious effects were reported: “hashish eases the muscles of the limbs, but it produces senseless talks”, and “if taken in excess it produces hallucinations and a staggering gait”. If taken for long periods of time it causes people to communicate with spirits [47]. Nonetheless, in some traditions, such as Indian medicine, the use of cannabis has persisted for centuries. Around 1840, William O’Shaughnessy, an Irish doctor working in India with the British Army, observed the proprieties of cannabis-derived drugs in the treatment of cramps, headaches, convulsions, neuralgia, sciatica, and tetanus [46]. The medical use of cannabis was then re-introduced in Europe, and experimental work suggested that the Indian claims about cannabis-based treatment were indeed likely spread [23].

The beginning of the 20th century saw the use of medicinal cannabis curtailed due to its chemical and physical properties which made the creation of standardized and reliable preparations impossible. In the same period, the development of synthetic fibers such as nylon led to a sharp decline in cannabis cultivation for textile purposes [48]. Although cannabis used by textile industries represents a variety without psychoactive properties, known as hemp, its application was significantly associated with marijuana. Indeed, in 1937 a US federal law, the Marijuana Tax Act, restricted the usage and cultivation of all cannabis, without distinction between hemp and marijuana. After that, given its popularization for recreational use around the world, cannabis was classified as a substance of abuse, and any application of the plant was prohibited [48,49,50]. 

The prohibition of cannabis also had a negative impact on scientific research. However, investigations into the chemistry and pharmacology of the plant did not completely stop, and the analysis of resin extracts allowed for the identification of several compounds. Among these, tetrahydrocannabinol (THC) was suspected to be the main psychoactive constituent of cannabis, but its structure was not fully characterized [51]. Only 20 years after this, the development of Nuclear Magnetic Resonance (NMR) spectroscopy allowed for the designation of Δ9-tetrahydrocannabino l [52]. This event opened new frontiers in the understanding of cannabis proprieties and its related neuronal substrates. Indeed, cannabis is the source of at least 66 compounds now known as cannabinoids [53]. CBN;, which is probably formed from THC during the conservation of harvested cannabis, was the first plant cannabinoids (phytocannabinoids) to be discovered at the end of the 19th century, and comes from a red oil extract of cannabis. CBN structures were determined in the early 1930s by R.S. Cahn, with its chemical synthesis first achieved in 1940 in the laboratories of R. Adams in the USA and Lord Todd in the UK. A second phytocannabinoid, (−) CBD, was first obtained from cannabis in the same year by Adams and colleagues, probably associated to cannabidiolic acid, while THCs were first extracted from cannabis in 1942 by Wollner, Matchett, Levine and Loewe, most likely as a mixture of (−)-Δ8- and (−)-Δ9-THC. Both THC and CBD are present in cannabis, mainly as decarboxylated acids upon the heating and combustion of cannabis. The structures and stereochemistry of CBD and Δ9-THC, naturally occurring as an (−)-enantiomer, were discovered by Raphael Mechoulam and colleagues, for CBD in 1963 and for Δ9-THC in 1964, respectively. It was also in Mechoulam’s laboratory, in 1965, that (±)-Δ9-THC and (±)-CBD were first synthesized, a development that was soon followed by the synthesis of the (+)- and (−)-enantiomers, both of these cannabinoids, and of Δ8-THC [54]. 

In the last decade, cannabis legislation has changed in several countries. A large number of countries around the world approved the legalization of medicinal cannabis and hemp (finally discerned from marijuana). Additionally, Uruguay, Canada, Georgia, Mexico, South Africa, and 18 US states legalized recreational cannabis consumption, and in many other countries, mostly in Asia, the use of cannabis has been decriminalized [55]. Globally, the public acceptance of legalizing cannabis and its medical application has increased, and therefore a better understanding of its plethora of effects on the human brain and body is of central interest.

After the THC discovery, extensive studies on the pharmacology and biochemistry of cannabis were carried out, with particular interest regarding the mode of action of THC and other cannabinoids of the plant. Two mechanisms were postulated: the first hypothesis was based on the lipophilic nature of cannabinoids, suggesting that they might act via the chemical interaction with biological membranes, modifying their proprieties; the second one suggested that cannabinoids might act through still undiscovered receptors, thereby modulating cellular signaling. This second hypothesis was based on the observation that THC acts by reducing the activity of adenylyl cyclase (AC), but only in particular cell types, indicating the specific and not ubiquitous action of THC, as expected for cannabinoid-induced membrane fluidity changes [56].

The development of synthetic cannabinoids helped to address this issue. Indeed, compounds such as CP-55,940, which is 10–100 times more potent in vivo than THC, allowed the autoradiography of cannabinoid-specific binding sites in brain sections from several mammalian species, including humans. The study revealed a specific and conserved labeling profile, suggesting the presence of a specific receptor [56,57].

At a conference of the National Academy of Science’s Institute of Medicine in 1990, Dr. Lisa Matsuda announced a fundamental discovery: the identification of the precise DNA sequence coding for THC-sensitive receptors. These were highly expressed in the brain, in accordance with the psychoactive effects of cannabinoids. The receptors were successfully cloned and were called Cannabinoid-type1 receptors (CB_1_ receptors [58]). Even though their physiological role was still a mystery, the high-level of expression and distribution of CB_1_ receptors suggested the existence of endogenous ligands. A few years after this, in 1992, an endogenous brain molecule binding to the same receptor that was sensitive to THC was identified. This substance, arachidonoylethanolamide was named Anandamide after the Sanskrit word “ananda” (bliss). Composed of ethanolamide and arachidonic acid, it represented the first discovered endogenous cannabinoid, or endocannabinoid (eCB; [59]). From this point, interest in research with regard to the field of cannabinoids notably increased, leading to several fundamental discoveries. Munro and colleagues, in 1993, identified a second receptor, CB_2_ which, different from CB_1_, is mainly expressed in peripheral cells and in the immune system [60]. Furthermore, a second endocannabinoid has been discovered, 2-AG, which, similarly to AEA, is a derivative of arachidonic acid [61]. Altogether, this evidence promoted the concept of the ECS, which participates in the regulation of physiological processes [62]. The ECS was then identified in several animal species, including Cnidaria, the first animal organism to have developed a neural network [63]. The high degree of evolutionary conservation of the ECS across the species suggest its importance in physiology, animal adaptation, and survival [64]. 

### 1.2. Biological Targets of CBD Action 

***GPCRs.*** As previously introduced, the binding of CBD to cannabinoid receptors is relatively weak [33,65], although one exception of a partial CBD agonism of human CB_2_ receptors in the heterologous system has been reported [65]. However, low concentrations of CBD are able to antagonize the effects of the CB_1_ and CB_2_ receptor agonists [66], a phenomenon explained by a negative allosteric receptor-modulation in recent pharmacological studies [33,65,67]. On the other hand, CBD’s main mechanisms of action have been proposed to be independent from the ECS. Several studies proposed that some of these effects seem be mediated by the serotoninergic 5HT1a receptor (5HT1a), which is coupled to the Gi protein (like CB_1_ and CB_2_). Indeed, although it has a relatively weak binding to 5HT1a, CBD showed a positive allosteric modulation of this receptor [68,69]. Various orphan GPCRs have also been proposed as targets for CBD. This drug antagonizes GPR55 [70], which is involved in actin cytoskeletal processes during movement and migration [71] (thanks to G_13_ alpha coupling). CBD is also an inverse agonist for GPR3, GPR6 and GPR12 [72], which can explain CBD’s actions involving cell survival, proliferation and neurite outgrowth, as well as concerning neuropathic pain [72]. Other Gi-coupled receptors have been proposed by many studies as binding sites for CBD, such as the μ- and δ-opioid, and high-affinity D2- and D3-dopamine receptors [73]. 

***Ionotropic Receptors.*** The physiological effects of CBD might largely be explained by its high affinity for inotropic receptors. CBD potently activates cationic channels belonging to the family of transient receptor potential (TRP) channels on different cell types (in particular TRPA1, TRPV1, TRPV2 and TRPV4 [74,75,76,77]]). Further ionotropic receptors negatively modulated by CBD are TRPM8, which is directly antagonized [78], and the α7 nicotinic acetylcholine receptor and the serotonin receptor 5HT3a [79,80] on which CBD acts as a negative allosteric modulator. A positive allosteric modulator of CBD has been observed for anionic ion channels, such as glycine (GlyRs) and GABA_A_ receptors [81,82]. CBD also acts on voltage-gated calcium Cav3.1/Cav3.2 and sodium channels Cav3.3, inhibiting cationic currents, as well as decreasing the conductance of voltage-dependent anion channel 1 (VDAC1) [83,84,85]. 

***Transporters.*** Several reports highlighted CBD interaction with intracellular transporters of endocannabinoids, with a direct inhibition of anandamide uptake, in particular fatty acid binding proteins (FABP) 1, 3, 5 and 7, resulting in the potentiation of ECs actions [86]. Indeed, CBD boosts AEA levels in rat brains in and in human serum via an N-acyl phosphatidylethanolamine phospholipase D dependent mechanism [87,88]. Another target for the anti-inflammatory and sedating effects of CBD is the blockade of adenosine uptake acting through the equilibrative nucleoside transporter (ENT) [89,90]. The ATP-binding cassette super-family G member 2 (ABCG2) or P-glycoprotein), and the multidrug resistance proteins (multidrug resistance-associated protein 1 (ABCC1) and Mg2+-ATPase also have shown to be modulated by CBD [91,92] (Figure 3).

***Enzymes.*** CBD also modulates the activity of several enzymes, including the members of the cytochrome P450 superfamily (CYPs), as their interaction with CBD may influence the clearance of various drugs, including non-steroidal anti-inflammatory ones. CBD inhibits various CYP superfamily members (CYP1B1, CYP2C19, CYP2C9, CYP3A4 and CYPC3A70) [93]. According to its structural properties, CBD might also interact with various lipid metabolism enzymes, and in particular AEA. Indeed, FAAH activity is inhibited by with discrepant results between the rat and human isoforms [74,75]. Interestingly, lipooxygenases (LOXs), are targets of CBD [94]. With regard to inflammatory processes, CBD always stimulates COX1 and COX2, while inhibiting phospholipase A2 (PLA2) [95,96]. At the mitochondrial level, CBD is able to inhibit, although with low potency, mitochondrial complex I, II and IV [97]. CBD also interferes with the serotonin to melatonin metabolism, as well as with the tryptophan catabolism, by acting on indoleamine-pyrrole 2,3-dioxygenase (IDO) [98,99]. CBD’s interaction with the enzymes involved in the steroid metabolism such as acyl-CoA cholesterin acyltransferase (ACAT) or testosterone hydroxylase, were examined in a few studies, although consistent results were not realized [100,101]. 

***Nuclear factors*.** In the context of inflammation, nuclear receptor peroxisome proliferator-activated receptor gamma (PPARγ) appears to be one of the most relevant targets of CBD. Indeed, CBD is a weak full agonist of this receptor [102]. Moreover, nuclear factor erythroid-derived 2-like 2 (Nrf2) in the activated microglia is also described as a putative culprit for alterations of inflammatory gene expression patterns by CBD [103].

***Inflammatory mediators.*** Mechanistically, CBD administration is known to suppress the immune response by impairing cytokine production and inflammation [104]. As previously mentioned, the CBD affinity for the CB_1_ and CB_2_ receptors is not very high, and thus its pharmacological activity might not be mediated by cannabinoid receptors. Instead, CBD exerts its anti-inflammatory actions by modulating the TRPV1 receptor, as shown by the blockage of CBD effects by TRPV1 antagonists [105,106]. A regulation of immune responses by the Janus kinase/signal transducers and the activators of the transcription (JAK/STAT) signaling pathway is also negatively modulated by CBD. Indeed, the triggering of JAK/STAT via the release of TNF-α, Interferon-γ (IFN-γ), Interleukin-1 (IL-1), IL-2, and the IL-6 inflammatory cytokine, is attenuated by CBD in vitro and in vivo [107]. CBD is also able to prevent the nucleotide-binding oligomerization domain-like receptors’ (NLR) inflammasome complex activation, involving the NF-κB, MAPK, and IFN pro-inflammatory pathways, thereby reducing pro-inflammatory cytokines, such as IL-1β and IL-18 [108]. Furthermore, in the inhibition of the rapid cellular uptake of adenosine by ENT [89], CBD has also been studied to modulate adenosine, inducing protective anti-inflammatory effects via the A_2A_ receptor [109]. Thus, during inflammation, the inhibition of intracellular adenosine uptake by CBD might promote a protective signaling mechanism [109]. In summary, understanding the mechanism behind the pharmacological reduction of inflammation by CBD will provide a strong rationale for the medical use of CBD as a novel therapeutic option for inflammatory diseases (Figure 4). 

### 1.3. CBD in Dentistry

#### 1.3.1. Oral Mucosa 

In the use of cannabinoids, the oral mucosa is the tissue that primarily comes into contact with them and interacts with them. Studying their physiological, therapeutic and non-therapeutic role in more detail and evaluating their effects is of significant importance.

CB_1_ and CB_2_ receptors have been detected on oral mucosal epithelial cells. They modulate their functions: CB_2_ receptors stimulate the proliferation and differentiation of human epithelial keratinocytes, while CB_1_ receptors have the opposite effect [18]. 

The cannabinoid receptors, CB_1_ and CB_2_, have also been identified at the level of the connective tissue of the *lamina propria*. However, the current scientific data regarding cannabinoids on receptors in oral mucosal tissues are still scarce [110]. Interestingly, CB_1_ and CB_2_ receptors are also present in the epithelial cells and taste buds of the tongue, where their function would appear to be regulated by the physiological-pathological conditions of the tongue [110]. One example is the presence of burning mouth syndrome, which is associated with a decrease of CB_1_ receptor expression, while the CB_2_ receptor expression increases. Furthermore, oncological conditions, such as mobile tongue squamous cell carcinoma, seems to involve the activation of ECS, since the expression of both CB_1_ and CB_2_ receptors has been shown to be increased [111,112]. There is little data on the presence of CB_1_ and CB_2_ receptors in dental pulp, where CB_1_ receptors have been detected in the sympathetic nerve fibers and on the surface of the pulp, bordering dentin [113]. This would suggest a possible therapeutic target against dental pain, although this possible role of cannabinoids requires further study. CB_1_s are also present on human odontoblasts, where they are hypothesized to respond to immune challenges [114,115]. Indeed, their activation and subsequent cyclic adenosine mono-phosphate (cAMP) signaling enables the TRPV1 mediated extracellular Ca(2+)ion passage (TRPV1) via extrusion Na(+)-Ca(2+) exchangers (NCXs), promoting the production of a secondary dentin bridge in response to odontoblast stimuli [116]. In the salivary glands, CB_1_ and CB_2_ receptors have specific localizations. In the major salivary glands, CB_1_ expression is found at the striatal duct cells, while CB_2_ is found in the acinar cells, especially in the myoepithelial cells, which are responsible for the secretion of saliva [117,118]. Interestingly, the presence and distribution of CB_1_s in salivary glands would appear to be regulated by the type and amount of food [119] and, furthermore, salivary secretions are modulated by both CB_1_ and CB_2_ receptors [120,121,122].

#### 1.3.2. Periodontal Tissue

The CB_1_ and CB_2_ receptors are expressed in the periodontium, and their distribution changes based on periodontal tissue conditions [42]. In a healthy periodontium, CB1s are more highly expressed in the periodontal ligament (PDL), and are more active in the epithelium than in the PDL [123,124]. Interestingly, the presence of bacteria increases the expression of CB_2_ receptors, whereas in a situation of sterile inflammation, both receptors are more highly expressed in PDL, but not in cementum and alveolar bone [125]. Thus, different expression patterns of the two receptors would appear to be related to different cellular activity (differentiation and proliferation), the control of inflammation, and the healing of the affected site [126]. Several reports seem to suggest a role for CB_2_ in periodontal tissue healing, especially in terms of modulating the migration and adhesion of periodontal cells upon input from the focal adhesion kinase (FAK) and mitogen-activated protein kinase (MAPK) systems [124,126,127,128]. 

Consistent beneficial effects of CBD have been described in vitro and in vivo, in addition to conventional periodontal therapy. Using the CBD analog HU-308, the authors found a role for the CB2 receptor in modulating the extent of periodontal damage and its impact on the gingival tissue, alveolar bone, and salivary function [129]. In the same study, CBD demonstrated anti-inflammatory and anti-bone resorption properties by inhibiting the RANK/RANKL system and reducing the levels of pro-inflammatory cytokines [129]. An alternative approach to periodontal therapy might be the CBD-mediated activation of gingival fibroblasts with repairing growth factors and/or the inhibition of metalloproteinases [130,131]. CBD has also been observed to attenuate bacterial inflammatory periodontal diseases thanks to its antimicrobial properties [132,133]. This drug might also be a suitable medicinal alternative in oral mucositis, given its anti-inflammatory properties, which can reduce the severity and extent of lesions [36,134] since CBD also promotes the curative process of common ulcers. Similarly to synthetic oral medicines, CBD is effective in reducing the bacterial charge in dental plaque [133]. Furthermore, CBD is endowed with biocompatibility and osteoinductivity [126,135] as it has been shown to promote fracture healing, possibly activating the p42/44 pathway in mesenchymal cells, which then differentiate into osteoblasts at the lesion site [73]. Thus, analgesic, anti-inflammatory, biological, antimicrobial, and osteoinductive properties of CBD might underlie its positive effects in dentistry, as suggested by most of the recent literature. This has paved the way for the development of patents for the implementing of CBD formulations in dentistry. 

## 2. Discussion

The subjects described in the studies on possible CBD use in dentistry, as well as the patents reviewed, which will be discussed in the following sections, are endodontic therapy, periodontology, oral medicine, and oral surgery, together with the oral heath potentials of CBD and the future research questions.

***Endodontic therapy (direct hooding).*** The exposure and subsequent bacterial contamination of exposed vital dental pulp as a result of trauma and deep caries is a treatment that if not well performed could lead to pulp inflammation, pain, and necrosis in immature teeth and to an arrest of the root maturation process, with the risk of loss of dental elements [136]. Thus, the induction of odontogenesis, using bioactive materials, would lead to the preservation of pulpal viability [137].

Given the expression of cannabinoid receptors in dental pulp, it is not surprising that cannabinoids (including CBD) induce odonto/osteogenic differentiation, stimulating the proliferation, migration and differentiation of dental pulp stem cells, producing increased collagen synthesis and mineralization, with protective effects on pulpal vitality in some studies [114,136,138]. Furthermore, CBD is also capable of inhibiting the action of TNF-α, which blocks stem cell differentiation, reduces the action of the pro-inflammatory cytokines TNF-α, interleukin (IL)-1β and IL-6 [139] and, by activating CB_1_ receptors, stimulates the extracellular Ca^2^+ entry inducing reparative dentin formation in odontoblasts [140,141].

By activating CB_2_ receptors and stimulating the Mitogen-Activated Protein Kinase (MAPK) pathway, CBD enhances the expression of angiogenic and odontogenic genes, such as Osteopontin (OPN), RUNX family transcription factor 2 (RUNX2), Vascular Endothelial Growth Factor-Based angiostatics (VEGFR1), the intercellular adhesion molecule 1 (ICAM-1), dentinal sialophosphoprotein (DSPP), the dentinal matrix acid phosphoprotein 1 (DMP-1), and the alkaline phosphatase (ALP) [136,142,143]. 

***Periodontal Therapy.*** In a pre-clinical model of periodontitis, CBD is able to prevent alveolar bone loss [131] thanks to its anti-inflammatory action. Indeed, the specific activation of CB2 receptors by CBD is endowed with analgesic and anti-inflammatory benefits, preventing any secondary effects due to CB_1_ receptor activation [34,104], since the activation of CB_2_ receptors physiologically protects periodontal tissues against excess inflammatory processes [131]. Furthermore, the antagonistic effect of CBD on NT-kB prevents the production of interleukins and other inflammatory mediators such as cytokines, chemokines, and pro-inflammatory growth factors [104]. This effect is often accompanied with reduced macrophage and neutrophil migration with less oxidative stress [104]. As for the ECs, the anti-inflammatory potential of CBD, whether directly or indirectly, act through cannabinoid receptors [34,104], and is often associated with increased gingival fibrosis, with CBD increasing the production of gingival fibroblasts [130] via the induction of transforming growth factor beta (TGFβ) levels [130]. Lastly, the CBD-induced increase in the levels of AEA, may also promote fibrosis via cannabinoid and other receptors [135].

***Oral Medicine.*** The anti-inflammatory and analgesic readouts of the administration of CBD are dose-dependent, without ideal doses having possible antioxidant and anti-inflammatory action [36,73,144]. This synergy between different CBD effects may render this drug more potent against pathological states such as oral mucositis compared to classical antioxidants [41,145]. Thus, CBD is a novel potential therapy for the treatment of symptoms characterizing this condition, improving epithelial changes in of ulcer lesions in vivo [41,145]. An important feature of CBD for generating this type of tissue response is the action on keratinocytes, with no undesirable effects. However, despite reducing the global inflammatory status, CBD did not accelerate wound healing [134], making the keratinocytes action of this drug controversial, and with the need to understand its mechanisms of action. The in vivo administration of the synthetic analog HU-308 prevented inflammation and alveolar bone loss in gingival tissues in a model of LPS periodontitis [129]. This effect, mediated by CB_2_ receptors, was mainly localized due to reduced osteoclastogenesis, indicating the CB_2_ receptor-mediated prevention of bone loss by targeting bone cells together with the inhibition of pro-reabsorption factors [129]. CBD is also effective as an antimicrobial agent. Indeed, high doses of CBD suppresses the growth of key bacterial components of subgingival microbiota in dental plaque [133]. Dental plaque is mainly composed of Gram-positive bacteria, which are susceptible targets for the well-known antimicrobial action of CBD mentioned above [30,139]. When compared with other oral hygiene products, CBD strikingly reduced the density in bacterial colonies as with other well-established oral hygiene formulations, with variations in efficacy due to the heterogeneity of oral biofilms [133].

***Traumatology/Surgery.*** As previously mentioned, CBD possess promising biological and osteoinductive properties. Alone or in combination with other drugs, CBD effectively and consistently improved the cell migration and bone differentiation migration of microglial cells via the ECS [131]. The ECS is present in both skeletal sympathetic nerves and bone cells, and cannabinoids play a key role in the homeostasis of bone mass [15]. In this scenario, CBD has been shown to stimulate the expression of the PLOD1 gene, showing an increased collagen maturation which triggers bone protein expression and mineralization [73,146]. These actions may results in neobone formation and the improvement of the biomechanical properties of bone tissue, making CBD a suitable therapeutical adjuvant for bone loss due to surgery or trauma. 

***Other comorbidities.*** CBD can be useful in patients with malignancies. Indeed, a frequent side effect of cancer-related chemotherapy and radiation, which kills both cancerous and healthy tissues, is oral mucositis. Despite the paucity of research, the antioxidant properties of CBD imply that it might be used to reduce the oxidative stress linked to oral mucositis [145]. Furthermore, the most upsetting side effects experienced by oncology patients receiving anticancer treatments are nausea and vomiting. This can lead to severe consequences in the mouth, as the acidity of gastric juice favors the alteration of the oral microbiota, with inflammation of the mucosa as well as the erosion of dental enamel. Vomiting and nausea continue to be particularly difficult to cure with present therapies, stressing the need for substitute approaches. In this scenario, CBD proved to be effective in reducing nausea and vomiting [147], and might attenuate all oral cavity related-damage [148]. Another application of CBD representing a possible benefit to dentistry is in the treatment of arthritis that often involves the temporomandibular joint. Indeed, CBD has shown potential beneficial effects in preclinical studies for reducing the inflammation and pain associated with arthritis [149]. For example, CBD inhibits T-cell proliferation, thus preventing the production of IFN- and TNF-, and the development of Th1-mediated autoimmune rheumatoid arthritis [150]. Primarily because of its anti-inflammatory and antioxidant qualities, CBD has a variety of advantageous effects in the context of hyperglycemia. Interestingly, type 2 diabetes and obesity have both been linked to the chronic overactivation of the ECS [151], which leads to the possibility that CBD could also be used therapeutically to treat type 2 diabetes [152]. Indeed, this pathological state has an important impact on oral health and in oral infections in particular that are brought on by diabetes [153]. Lastly, CBD has also been shown to be effective in treating epilepsy according to randomised, placebo-controlled research that showed a significant reduction in seizure frequency [154,155]. This CBD effect might also lead to beneficial effects on oral health, as it reduces the risk of trauma and injuries to the lip and oral tissues that often occur during epileptic seizures [156]. 

***Current scientific and technological knowledge.*** According to the recent studies on the actions of CBD in the mouth, there is growing attention to the technological exploitation and appropriation relative to the implementation of CBD preparations in dentistry. However, only one patent [157], which was published in 2018, was accompanied by a scientific study [130] in 2020, despite the increasing progress for this kind of development. The patents registered for applying CBD in dentistry are mostly related to oral care formulations rather than for its use for oral diseases. Thus, given the modern tendency towards the use of natural products [158], there is plenty of space for CBD exploration for potential dentistry-related applications. Furthermore, thanks to the current low cost of these plant derived compounds, CBD and synthetic analogs are promising drugs for several diseases [159]. However, some limitations can also be highlighted. First, there is no clear governmental regulation for the quality of CBD [160], which could result in different sources of its manufacture, leading to the variable efficiency of CBD preparations. Second, the components of plant-derived natural products vary greatly because of the heterogeneity in environmental conditions, making the quality and the levels of natural products reliant upon several conditions, such as vegetation, geographic location, and extraction conditions [161]. Therefore, a natural preparation may contain different chemicals that influence the therapeutic activity. Second, there is no standardized practice with regard to analytical techniques characterizing the products. Thus, changes in the control of these materials’ origin, storage conditions, and production and possible contaminations are all factors to keep in mind with regard to the value of products and their therapeutic effects [162].

## 3. Conclusions

Despite the growing interest of CBD use for medical purposes, little scientific literature and few patents exist with regard to its application in oral health. Studies on the effects of cannabis use on oral tissues and oral health are irrelevant. Research evidence so far promotes CBD as an analgesic, antimicrobial, anti-inflammatory, and osteoinductive drug with potential applications in periodontal and dentistry applications, although the available patents rely on compositions for oral care products such as toothpaste, mouthwash, and dental floss. There is significant interest in *C. sativa* due to the growing medical and public interest in its use for multiple conditions. CBD approval for the treatment of rare epileptic conditions, for which the US Drug Enforcement Administration (DEA) changed its status to Schedule V (low abuse potential), has expanded CBD’s research potential. Since cannabinoids are clinically relevant in terms of reducing pain symptoms, they represent a potential avenue for pharmacotherapy with regard to opioid abuse and related deaths. The pharmacological effects of pure CBD have been studied extensively, with low adverse effects seen in both preclinical and clinical settings. There are currently several CBD products (unapproved) that are sold without any standardization of CBD or other constituents that claim unproven health effects. Although cannabis-derived products exhibit anti-inflammatory properties, it remains unclear how well hemp/CBD products can replace traditional pain management, or if these products will work in tandem with existing therapies. At this point, CBD-rich oils are considered safe, but CBD’s reported side effects and drug interactions are not negligible, and must be considered before therapeutic recommendation. As a healthcare professional, it is important to understand that just as common medications are not suitable for every individual, neither are CBD products. However, since CBD’s efficacy for seizures has been proven, it is likely that physicians who are comfortable with using CBD will recommend its off-label use for other conditions. Thus, the current shortcomings in understanding the benefits of CBD-rich hemp oils have limitations due to the pharmacological and clinical effects not being predictable, in addition to the profiles of the marketed products varying greatly in terms of phytocompounds.

## Data Availability

Not applicable.

## Abbreviations

Δ9-THC	Δ9tetrahydrocannabinol
CBD	cannabidiol
ECS	endocannabinoid system
GPCR	G-protein coupled cannabinoid receptors
AEA	anandamide
2AG	2-arachidonoylglycerol
FAAH	fatty acid amide hydrolase
MAGL	monoacylglycerol lipase
5HT1a	5HT1a receptor
TRP	transient receptor potential
GlyRs	glycine
FABP	fatty acid binding proteins
ENT	equilibrative nucleoside transporter
ABCG2	ATP-binding cassette super-family G member 2
LOXs	Lipooxygenases
PLA2	phospholipase A2
IDO	indoleamine-pyrrole 2,3-dioxygenase
ACAT	acyl-CoA cholesterin acyltransferase
PPARγ	peroxisome proliferator-activated receptor gamma
Nrf2	nuclear factor erythroid-derived 2-like 2
IFN- γ	Interferon-γ
IL-1	Interleukin-1
NLR	nucleotide-binding oligomerization domain-like receptors
TGFβ	transforming growth factor beta
